# Development and validation of a machine learning-based ^18^F-fluorodeoxyglucose PET/CT radiomics signature for predicting gastric cancer survival

**DOI:** 10.1186/s40644-024-00741-4

**Published:** 2024-07-30

**Authors:** Huaiqing Zhi, Yilan Xiang, Chenbin Chen, Weiteng Zhang, Jie Lin, Zekan Gao, Qingzheng Shen, Jiancan Shao, Xinxin Yang, Yunjun Yang, Xiaodong Chen, Jingwei Zheng, Mingdong Lu, Bujian Pan, Qiantong Dong, Xian Shen, Chunxue Ma

**Affiliations:** 1 Department of Gastrointestinal Surgery Nursing Unit, Ward 443, The First Affiliated Hospital of Wenzhou Medical University, Wenzhou, Zhejiang Province China; 2 Department of General Surgery, The First Affiliated Hospital of Wenzhou Medical University, Wenzhou, Zhejiang Province China; 3 Department of Radiology, The First Affiliated Hospital of Wenzhou Medical University, Wenzhou, Zhejiang Province China; 4 Department of PET/CT, The First Affiliated Hospital of Wenzhou Medical University, Wenzhou, Zhejiang Province China; 5 Department of General Surgery, The Second Affiliated Hospital & Yuying Children’s Hospital of Wenzhou Medical University, Wenzhou, Zhejiang Province China; 6 Zhejiang Key Laboratory of Intelligent Cancer Biomarker Discovery and Translation, The First Affiliated Hospital of Wenzhou Medical University, Wenzhou, Zhejiang Province China

**Keywords:** Radiomics, Machine learning, Gastric cancer, Clinical tumor-node-metastasis, Positron emission tomography

## Abstract

**Background:**

Survival prognosis of patients with gastric cancer (GC) often influences physicians’ choice of their follow-up treatment. This study aimed to develop a positron emission tomography (PET)-based radiomics model combined with clinical tumor-node-metastasis (TNM) staging to predict overall survival (OS) in patients with GC.

**Methods:**

We reviewed the clinical information of a total of 327 patients with pathological confirmation of GC undergoing 18 F-fluorodeoxyglucose (18 F-FDG) PET scans. The patients were randomly classified into training (*n* = 229) and validation (*n* = 98) cohorts. We extracted 171 PET radiomics features from the PET images and determined the PET radiomics scores (RS) using the least absolute shrinkage and selection operator (LASSO) and random survival forest (RSF). A radiomics model, including PET RS and clinical TNM staging, was constructed to predict the OS of patients with GC. This model was evaluated for discrimination, calibration, and clinical usefulness.

**Results:**

On multivariate COX regression analysis, the difference between age, carcinoembryonic antigen (CEA), clinical TNM, and PET RS in GC patients was statistically significant (*p* < 0.05). A radiomics model was developed based on the results of COX regression. The model had the Harrell’s concordance index (C-index) of 0.817 in the training cohort and 0.707 in the validation cohort and performed better than a single clinical model and a model with clinical features combined with clinical TNM staging. Further analyses showed higher PET RS in patients who were older (*p* < 0.001) and those who had elevated CEA (*p* < 0.001) and higher clinical TNM (*p* < 0.001). At different clinical TNM stages, a higher PET RS was associated with a worse survival prognosis.

**Conclusions:**

Radiomics models based on PET RS, clinical TNM, and clinical features may provide new tools for predicting OS in patients with GC.

## Introduction

GC is the fifth most common cancer in the world, and East Asia continues to have a high incidence of the disease [1]. GC is usually asymptomatic in its early stages. hence, it often remains undiagnosed until it reaches an advanced stage. Comprehensive surgery-based treatment remains the primary approach for advanced GC management [2]. Although the 5-year overall survival rate of GC patients has improved recently and is higher than before [3, 4], predictive models and scoring tools for the prognosis of patients with GC are essential to improve individualized treatment. These tools can provide clinicians with follow-up treatment options to improve patient survival.

^18^F-FDG PET/CT imaging is a vital tool for the characterization, staging, and detection of distant metastases in patients with malignant tumors, including GC [5–7]. Particularly, this machine has a superior diagnostic ability for detecting distant metastases from cancer compared to computed tomography (CT) [8]. Parameters such as the maximum standardized intake value (SUV), total lesion glycolysis (TLG), and metabolic tumor volume (MTV) are often used to evaluate the prognosis of GC patients [9]. However, the spatial information of diagnostic images has not been fully analyzed and still relies on the rich experience and subjectivity of doctors.

Radiomics is an innovative technique that involves extracting high-dimensional information from standard medical images and delving deeply into hidden information regarding potential diseases that may not be visible to the human eye [10–13]. This holds tremendous potential for the diagnosis, prognostic assessment, and treatment prediction of GC, offering new opportunities in the field of precision medicine [14–17]. However, the number of radiomics tools based on PET imaging to predict survival models in patients is still limited in GC compared to other types of cancers [18–20].

Therefore, in this study, we attempted to extract image features from PET images to establish a relevant model that could be combined with the clinical TNM staging for patients with GC to determine whether the prognosis of patients with GC can be improved.

## Materials and methods

### Patient population

**Fig. 1:**
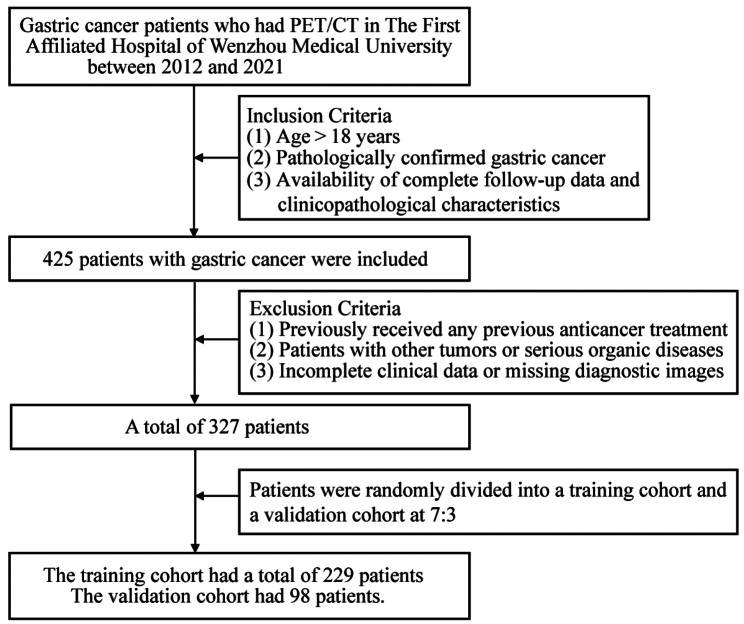
Patient enrollment process

A retrospective review of the medical records and imaging data of patients with GC who underwent ^18^F-FDG PET/CT at the First Hospital of Wenzhou Medical University between January 2012 and June 2021 was conducted. The inclusion criteria were age > 18 years, pathologically confirmed GC, and availability of complete follow-up data and clinicopathological characteristics. The exclusion criteria were GC patients who had previously received any previous anticancer treatment, patients with other tumors or serious organic diseases, and patients with incomplete clinical data or missing diagnostic images. The study ultimately recruited 327 cancer patients who were randomized 7:3 into the training and validation cohorts. A flowchart of patient screening is shown in Fig. 1. Clinical and pathological data of the patients, including sex, age, Nutritional Risk Screening 2002 (NRS 2002), body mass index (BMI), chemotherapy, CEA, Carbohydrate antigen199 (CA199), surgery, and clinical TNM staging, were retrospectively collected from medical records. Clinical TNM staging was determined by a radiologist and general surgeon according to the 8th edition of the American Joint Committee on Cancer staging system [21]. Each patient was followed up regularly. During the first 2 years, patients were monitored every 3 months and then every 6 months through outpatient treatment. OS was defined as the time to death from any cause and was used as the endpoint.

### PET/CT image acquisition

GC patients who underwent ^18^F-FDG PET/CT were imaged after a 6-hour fast, and blood glucose levels were maintained below 110 ml/dl. Patients were injected intravenously with 18F-FDG (3.7 MBq/kg) and imaged 60 min later using a ^18^F-FDG PET/CT scanner. (GEMINI TF 64, Philips, The Netherlands). The parameter settings were as follows: matrix size, 144 × 144; slice thickness, 5 mm; field of view, 576 mm; and emission scan time, 1.5 min for each bed position.

### PET/CT image segmentation and feature extraction

LIFEX software tools were used for volume of interest (VOI) delineation and feature extraction from each patient’s PET images [22]. Initially, a radiologist and a general surgeon drew the VOI segmentation using the Digital Imaging and Communications in Medicine protocol; then, an experienced radiologist checked this to ensure the accuracy of subsequent analyses. Two weeks later, the radiologists selected 50 patients and again segmented their VOI for assessment of VOI image quality. The LIFEX software program automatically measured the SUVmax of the segmented VOI for the target gastric lesions and selected the VOI using a 40% SUVmax threshold. Additionally, it automatically measured the MTV and TLG of target gastric lesions.

LIFEX software was used to extract 171 radiological features from tumor image VOI. The radiomics features were first-order statistics, shape-based features, gray-level co-occurrence matrix, gray-level run length matrix, gray-level size zone matrix, and neighborhood gray tone difference matrix.

### Construction of PET radiomics scores

To assess the consistency of the features and feature screening, we processed the extracted features. First, two radiologists calculated intraclass correlation coefficients (ICC) for the radiomics features extracted from the segmentation of 50 patients. High consistent features were defined as those with ICC values > 0.75. All high consistent features from the patients were standardized using the mean and standard deviation with the z-score algorithm.

Next, we used the LASSO with ten-fold cross-validation for feature selection in the training cohort; the features were ranked using the RSF method based on their importance and data predictive ability to obtain radiomics scores, called PET RS. Further validation was performed on clinical data of patients in the validation cohort. Based on this model, the PET RS for each patient was calculated for the validation cohort. The median PET RS (60) of the training cohort was used to classify all patients into high-risk and low-risk groups in order to reduce the distributional differences between the training and validation cohorts.

### Development and validation of predictive model

**Fig. 2:**
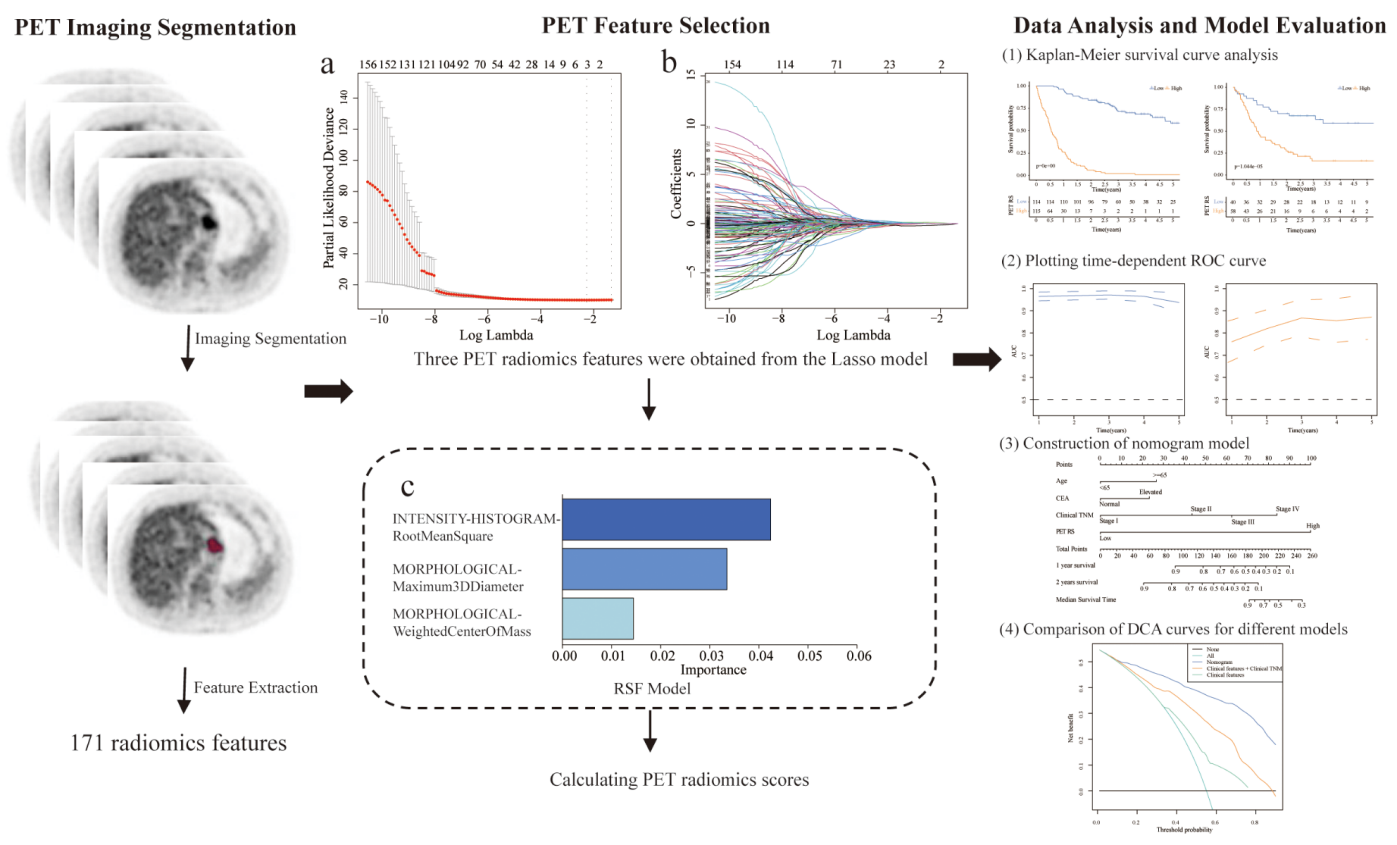
The workflow of the study. (**a**) Partialiegel Rlihood deviation values with respect to different λ values in the LASSO model. (**b**) Select the optimal λ value. (**c**) Rank the importance of each feature in the RSF model. PET: Positron emission tomography; LASSO: Least absolute shrinkage and selection operator; RSF: Random survival forest

For further analysis of the clinical features and PET RS of the patients, we performed univariate and multivariate Cox regression analyses to identify prognostically relevant clinical features. Subsequently, we selected features with *p* < 0.05 and constructed a nomogram that included clinical features, clinical TNM staging, and PET RS to visualize the results. Additionally, we constructed two other models, one with only clinical features and the other with clinical features combined with clinical TNM staging data, and used the C-index to assess the discrimination between the three models. A decision curve analysis (DCA) was performed to assess the clinical value of the nomograms [23]. The workflow is illustrated in Fig. 2.

### Statistical analysis

The R (version 4.3.3, https://www.r-project.org/) was used for statistical analysis. The t-test was used for normally distributed continuous data, and the Mann-Whitney U test for non-normally distributed continuous data. The chi-square test or Fisher’s exact test was used for categorical data. Independent prognostic factors that influenced outcomes were identified using univariate and multivariate Cox analyses. The Kaplan–Meier method was employed to construct survival curves, with the log-rank test subsequently employed to compare differences between the two cohorts. Nomograms, calibration, and DCA plots were generated using the R package. Statistical significance was set at *p* < 0.05.

## Results

### Patient characteristics

A total of 327 GC patients with were randomised to training (*n* = 229) and validation (*n* = 98) cohorts in a 7:3 ratio. The training cohort included 171 males and 58 females, and the validation cohort included 72 males and 26 females. Each patient was followed for at least two years, with 215 (65.7%) deaths and the median survival time of 19 months. Table 1 summarizes the detailed characteristics of the patients in the two cohorts.

**Table 1:** Clinical features of patients according to the PET RS in the training and validation cohorts

	Developing cohort				Validation cohort			
	All	Low	High	*P*	All	Low	High	*p*
	*N* = 229	*N* = 114	*N* = 115		*N* = 98	*N* = 40	*N* = 58	
Age				0.009*				0.008*
<65	72 (31.4%)	50 (39.5%)	27 (23.5%)		43 (43.9%)	24 (60.0%)	19 (32.8%)	
≥65	157 (68.6%)	64 (60.5%)	88 (76.5%)		55 (56.1%)	16 (40.0%)	39 (67.2%)	
Gender				0.119				0.857
Female	58 (25.3%)	34 (29.8%)	24 (20.9%)		26 (26.5%)	11 (27.5%)	15 (25.9%)	
Male	171 (74.7%)	80 (70.2%)	91 (79.1%)		72 (73.5%)	29 (72.5%)	43 (74.1%)	
BMI				0.342				0.639
Low	37 (16.2%)	15 (13.2%)	22 (19.1%)		13 (13.3%)	6 (15.0%)	7 (12.1%)	
Normal	165 (72.1%)	87 (76.3%)	78 (67.8%)		66 (67.3%)	28 (70.0%)	38 (65.5%)	
High	27 (11.8%)	12 (10.5%)	15 (13.0%)		19 (19.4%)	6 (15.0%)	13 (22.4%)	
NRS2002				0.015*				0.670
<3	162 (70.7%)	89 (78.1%)	73 (63.5%)		78 (79.6%)	31 (77.5%)	47 (81.0%)	
≥3	67 (29.3%)	25 (21.9%)	42 (36.5%)		20 (20.4%)	9 (22.5%)	11 (19.0%)	
Chemotherapy				0.090				0.581
No	145 (63.3%)	66 (57.9%)	79 (68.7%)		63 (64.3%)	27 (67.5%)	36 (62.1%)	
Yes	84 (36.7%)	48 (42.1%)	36 (31.3%)		35 (35.7%)	13 (32.5%)	22 (37.9%)	
CEA				0.001*				0.642
Normal	141 (61.6%)	82 (71.9%)	59 (51.3%)		66 (67.3%)	28 (70.0%)	38 (65.5%)	
Elevated	88 (38.4%)	32 (28.1%)	56 (48.7%)		32 (32.7%)	12 (30.0%)	20 (34.5%)	
CA199				< 0.001*				0.014*
Normal	163 (71.2%)	99 (86.8%)	64 (55.7%)		70 (71.4%)	34 (85.0%)	36 (62.1%)	
Elevated	66 (28.8%)	15 (13.2%)	51 (44.3%)		28 (28.6%)	6 (15.0%)	22 (37.9%)	
Surgery				< 0.001*				0.013*
No	93 (40.6%)	17 (14.9%)	76 (66.1%)		39 (39.8%)	10 (25.0%)	29 (50.0%)	
Yes	136 (59.4%)	97 (85.1%)	39 (33.9%)		59 (60.2%)	30 (75.0%)	29 (50.0%)	
Clinical TNM				< 0.001*				0.416
Stage I	26 (11.4%)	25 (21.9%)	1 (0.87%)		8 (8.16%)	5 (12.5%)	3 (5.17%)	
Stage II	34 (14.8%)	28 (24.6%)	6 (5.22%)		21 (21.4%)	7 (17.5%)	14 (24.1%)	
Stage III	69 (30.1%)	36 (31.6%)	33 (28.7%)		32 (32.7%)	15 (37.5%)	17 (29.3%)	
Stage IV	100 (43.7%)	25 (21.9%)	75 (65.2%)		37 (37.8%)	13 (32.5%)	24 (41.4%)	
SUV_mean_^†^	4.37 [3.14;6.57]	3.81 [2.72;6.11]	4.83 [3.37;7.23]	0.009*	4.52 [3.02;7.13]	3.92 [2.85;7.13]	4.99 [3.39;7.13]	0.167
SUV_max_^†^	7.22 [4.68;11.1]	6.55 [4.08;10.3]	8.23 [5.10;12.0]	0.004*	7.34 [4.52;12.1]	6.23 [4.10;12.1]	8.73 [5.36;12.1]	0.061
MTV^†^	17.7 [8.33;31.6]	11.5 [6.06;21.5]	23.6 [15.5;40.2]	< 0.001*	19.7 [8.46;33.8]	9.18 [7.24;16.9]	26.4 [18.8;43.6]	< 0.001*
TLG^†^	83.7 [31.9;160]	43.9 [20.8;117]	110 [64.4;221]	< 0.001*	90.0 [33.1;200]	37.2 [23.2;116]	124 [66.8;205]	< 0.001*

Note: Unless otherwise stated, data are numbers of patients and percentages are in parentheses. *, *P* < 0.05; ^†^, Data are median with interquartile range in parentheses; BMI: Body mass index; NRS 2002: Nutritional Risk Screening 2002; CEA: Carcinoembryonic antigen; CA199: Carbohydrate antigen199; TNM: Tumor-node-metastasis; SUVmean: Mean standardized intake value; SUVmax: Maximum standardized intake value; MTV: Metabolic tumor volume; TLG: Total pathological glycolysis

### Radiomics feature selection and PET radiomics scores building

**Fig. 3:**
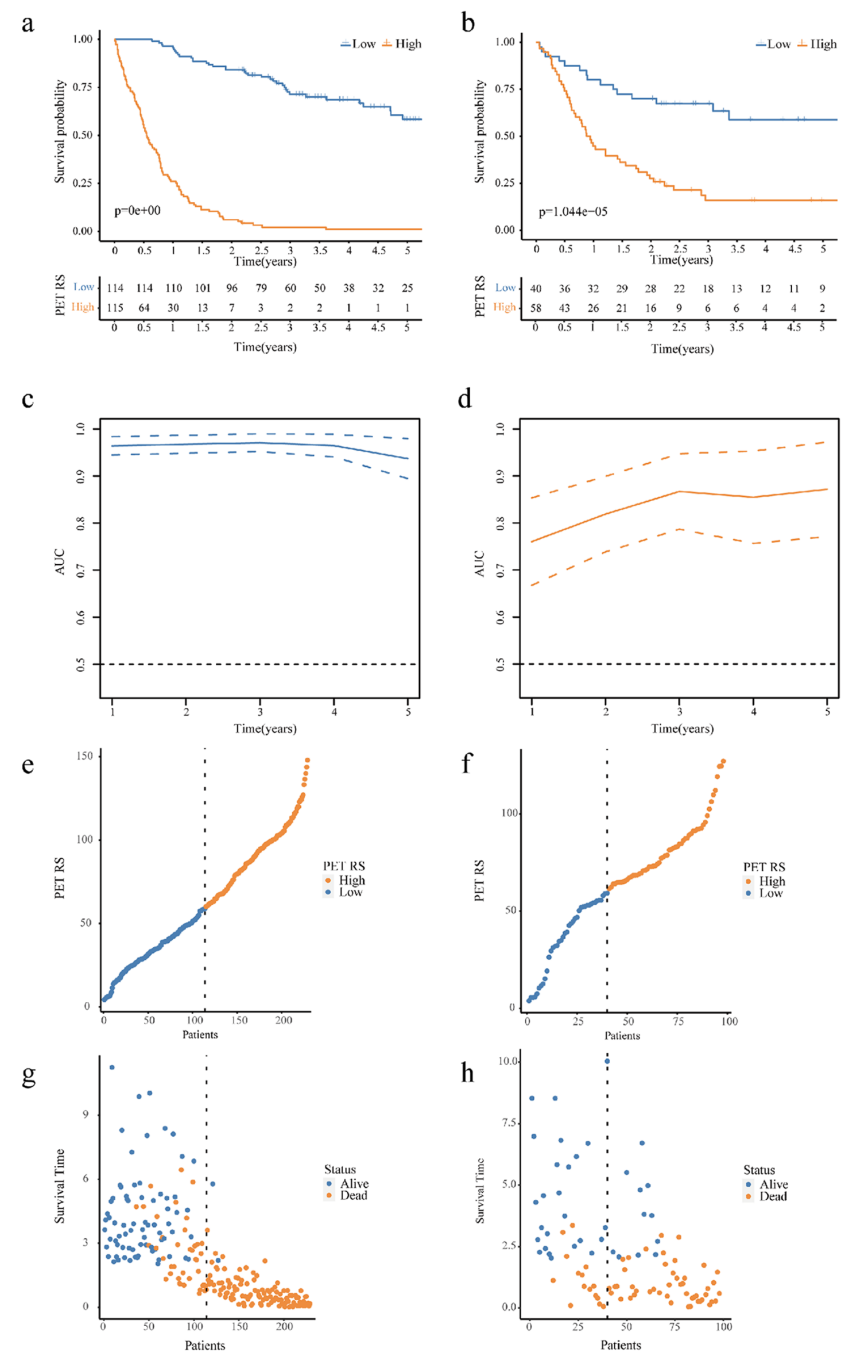
PET RS as a prognostic indicator. (**a**) Kaplan-Meier curves of OS between high- and low-PET RS groups in the training cohort. (**b**) Kaplan-Meier curves of OS between high- and low-PET RS groups in the validation cohort. (**c**) ROC curve of the PET RS in the training cohort. (**d**) ROC curve of the PET RS in the validation cohort. (**e, g**) The survival distribution of GC patients with different PET RS in the training cohort. (**f, h**) The survival distribution of GC patients with different PET RS in the validation cohort. PET: Positron emission tomography; RS: Radiomics score; OS: Overall survival; ROC: Receiver operator characteristics; GC: Gastric cancer

Lifex software extracted 171 PET radiomics features from the PET images of each GC patient. These characteristics were analyzed according to the following steps. Initially, according to the standard that the ICC value should be greater than 0.75, 160 features with high consistency were selected for model construction. We then used LASSO analysis to obtain the optimum value and three PET radiomics features with nonzero LASSO coefficients (Fig. 2a and b). Next, the selected features were further modeled based on the optimal iteration times of the RSF (Fig. 2c). Based on the PET RS calculated using this model, each patient was classified into high- and low-PET RS groups. We conducted survival analysis using the Kaplan–Meier survival curve and log-rank test, which showed significant differences between the high-PET RS group and low-PET RS group in the training (log-rank *p* < 0.001, Fig. 3a) and validation cohorts (log-rank *p* < 0.001, Fig. 3b). We further evaluated the accuracy of PET RS in the two cohorts for OS prediction using time-dependent receiver operator characteristics (Fig. 3c, d). Figure 3e-h depict the correlation between PET RS and survival outcomes, including survival status and time, for each patient.

### Model construction and evaluation

**Fig. 4:**
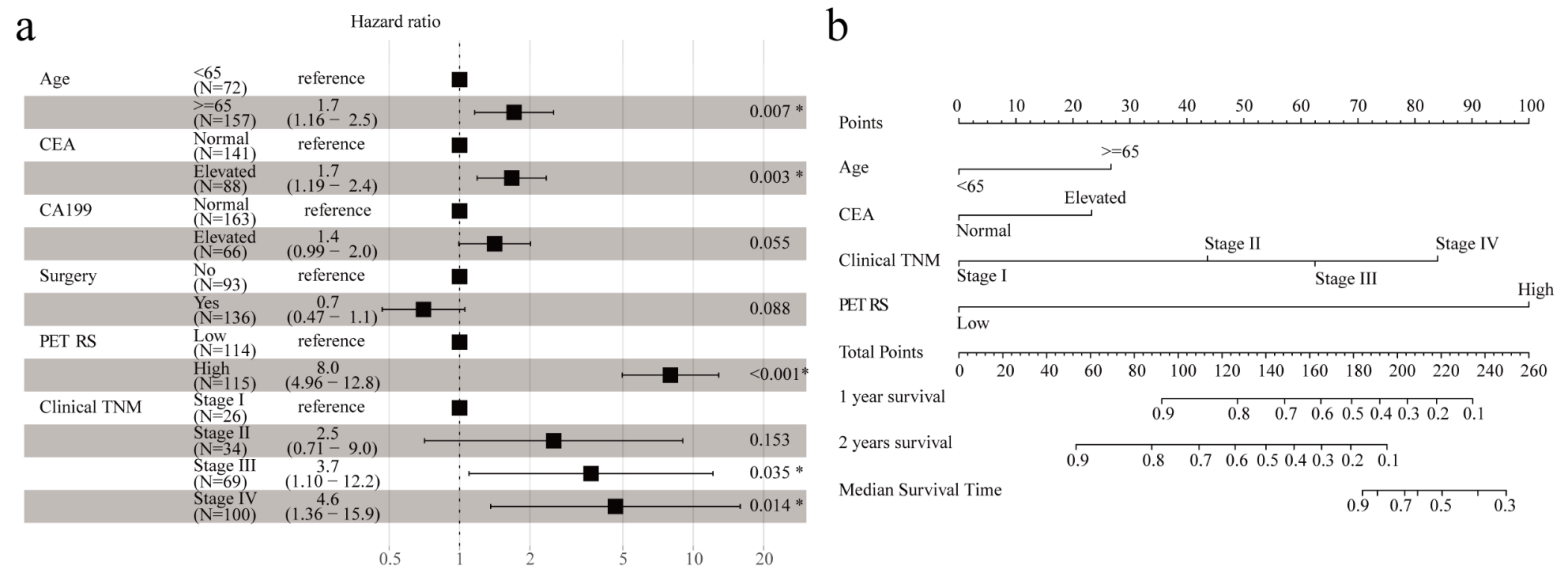
PET RS and different clinical features were used to predict OS. (**a**) Multivariate Cox analysis of PET RS and different clinical features in the training cohort. (**b**) Nomograms constructed based on PET RS and different clinical characteristics in the training cohort. PET: Positron emission tomography; RS: Radiomics score; OS: Overall survival; CEA: Carcinoembryonic antigen; CA199: Carbohydrate antigen199; TNM: Tumor-node-metastasis. *, *P* < 0.05

**Fig. 5:**
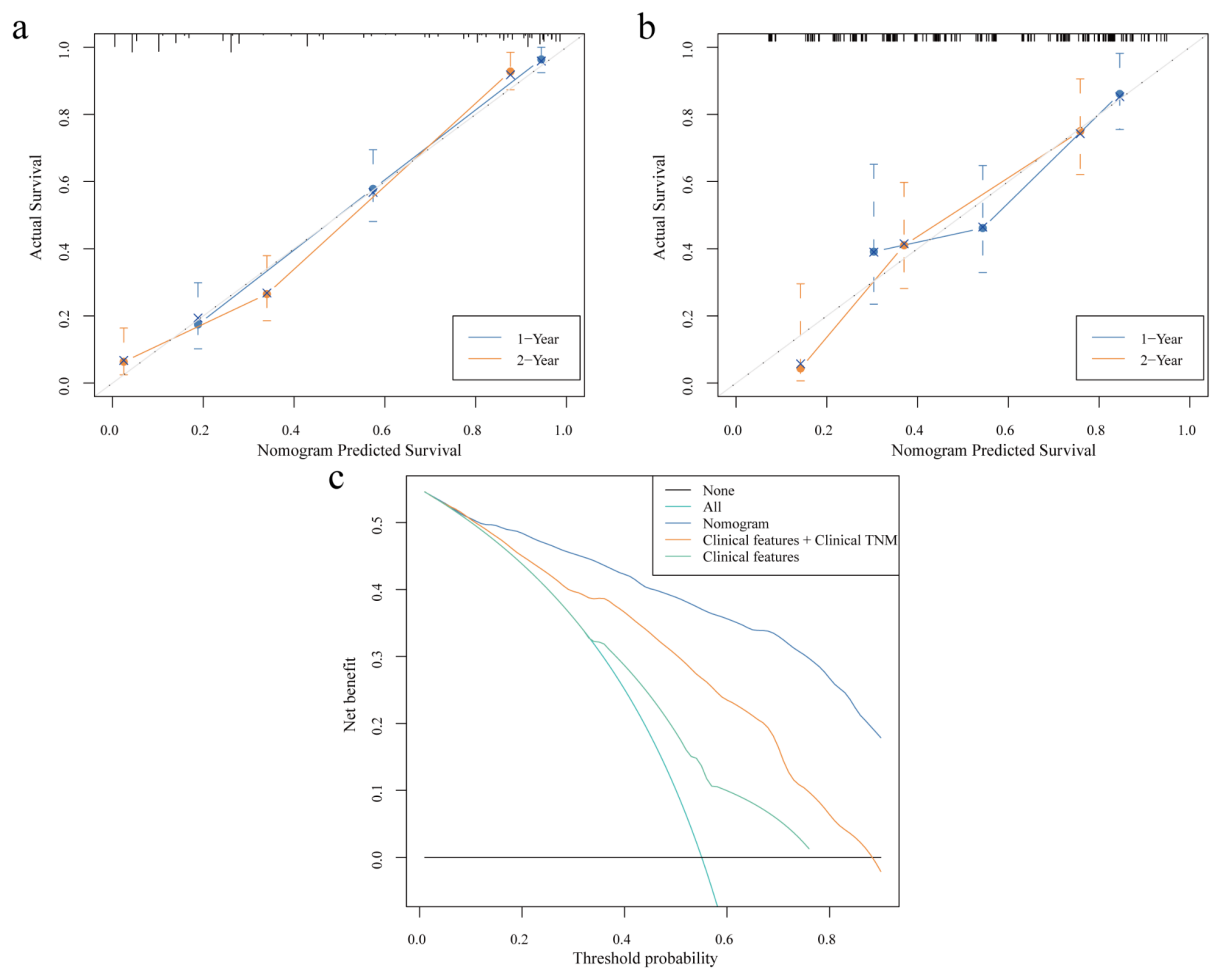
The nomogram correction curve. This shows consistent correction of 1-year and 2-year OS predictions in the training cohort (**a**) and validation cohort (**b**). Decision curves were analyzed for each model in all patients with GC to show the survival benefit (**C**). OS: Overall survival; GC: Gastric cancer; TNM: Tumor-node-metastasis

To comprehensively evaluate the impact of PET RS and other clinical features on prognosis, we performed a univariate Cox regression analysis in the training cohort. For features with *p* < 0.05 in univariate analysis, multivariate Cox regression analysis was performed (Table 2), showing that independent risk factors for OS were age, CEA, clinical TNM stage and PET RS (Fig. 4a). Additionally, to provide clinicians with a practical tool for risk assessment and therapeutic decision support, we built a nomogram based on the significant variables (age, CEA, clinical TNM stage, and PET RS) in the multivariate COX regression results (Fig. 4b). The nomogram C-index was 0.817 [95% CI: 0.790–0.844] for OS in the training cohort and 0.707 [95% CI: 0.640–0.774] for OS in the validation cohort. In both cohorts, the 1-year and 2-year nomogram calibration curves showed good agreement between the estimates and actual observations (Fig. 5a, b). Moreover, we developed two other models: a single clinical features model and a model of clinical features combined with clinical TNM staging characteristics. However, the nomogram’s C-index and the integrated Brier score (IBS) were better than those of the other two models (Table 3). The DCA curves indicated that within a reasonable range of threshold probabilities, the nomogram provided more beneficial prognostic information for patients with GC (Fig. 5c).

**Table 2:** Univariate and multivariate Cox analyses for OS of GC patients in the training cohort

Variables	Univariate Cox analysis				Multivariate Cox analysis			
	HR	HR.95 L	HR.95 H	*P*	HR	HR.95 L	HR.95 H	*P*
Age (> 65 vs. ≤65)	1.83	1.26	2.63	0.001*	1.71	1.16	2.53	0.007*
Gender (Female vs. Male)	1.17	0.80	1.69	0.417	-	-	-	-
BMI (Low vs. Normal)	1.47	0.98	2.23	0.064	-	-	-	-
BMI (High vs. Normal)	1.26	0.76	2.07	0.371	-	-	-	-
NRS2002(≥ 3 vs. <3)	1.41	0.81	2.45	0.219	-	-	-	-
Chemotherapy (Yes vs. No)	0.79	0.57	1.10	0.169	-	-	-	-
CA199 (Elevated vs. Normal)	2.86	2.05	3.99	*P* < 0.001*	1.41	0.99	2.01	0.055
CEA (Elevated vs. Normal)	2.08	1.51	2.86	*P* < 0.001*	1.67	1.19	2.35	0.003*
Surgery (Yes vs. No)	0.22	0.16	0.30	*P* < 0.001*	0.70	0.47	1.05	0.088
Clinical TNM (stage II vs. stage I)	3.58	1.01	12.70	0.048*	2.53	0.71	9.02	0.153
Clinical TNM (stage III vs. stage I)	9.02	2.80	29.02	*P* < 0.001*	3.65	1.10	12.15	0.035*
Clinical TNM (stage IV vs. stage I)	20.27	6.39	64.36	*P* < 0.001*	4.46	1.36	15.88	0.014*
PET RS	13.78	9.06	20.94	*P* < 0.001*	7.99	4.96	12.85	*P* < 0.001*

*, *P* < 0.05; HR: Hazard ratio; L: Low; H: high; BMI: Body mass index; NRS 2002: Nutritional Risk Screening 2002; CA199: Carbohydrate antigen199; CEA: Carcinoembryonic antigen; TNM: Tumor-node-metastasis; PET RS: positron emission tomography radiomics scores

**Table 3:** OS prediction performance of the three models in the training and the validation cohorts

Model	C-Index (95% CI)	IBS
**Training cohort**		
Nomogram	0.817(0.790–0.844)	0.089
Clinical features + Clinical TNM	0.730(0.691–0.769)	0.116
Clinical features	0.630(0.587–0.673)	0.165
**Validation cohort**		
Nomogram	0.707(0.640–0.774)	0.122
Clinical features + Clinical TNM	0.675(0.641–0.709)	0.152
Clinical features	0.582(0.509–0.656)	0.167

C-index, The Harrell consistency index; CI: Confidence interval; IBS: Integrated Brier score

### Analysis of PET RS and associated clinical features

**Fig. 6:**
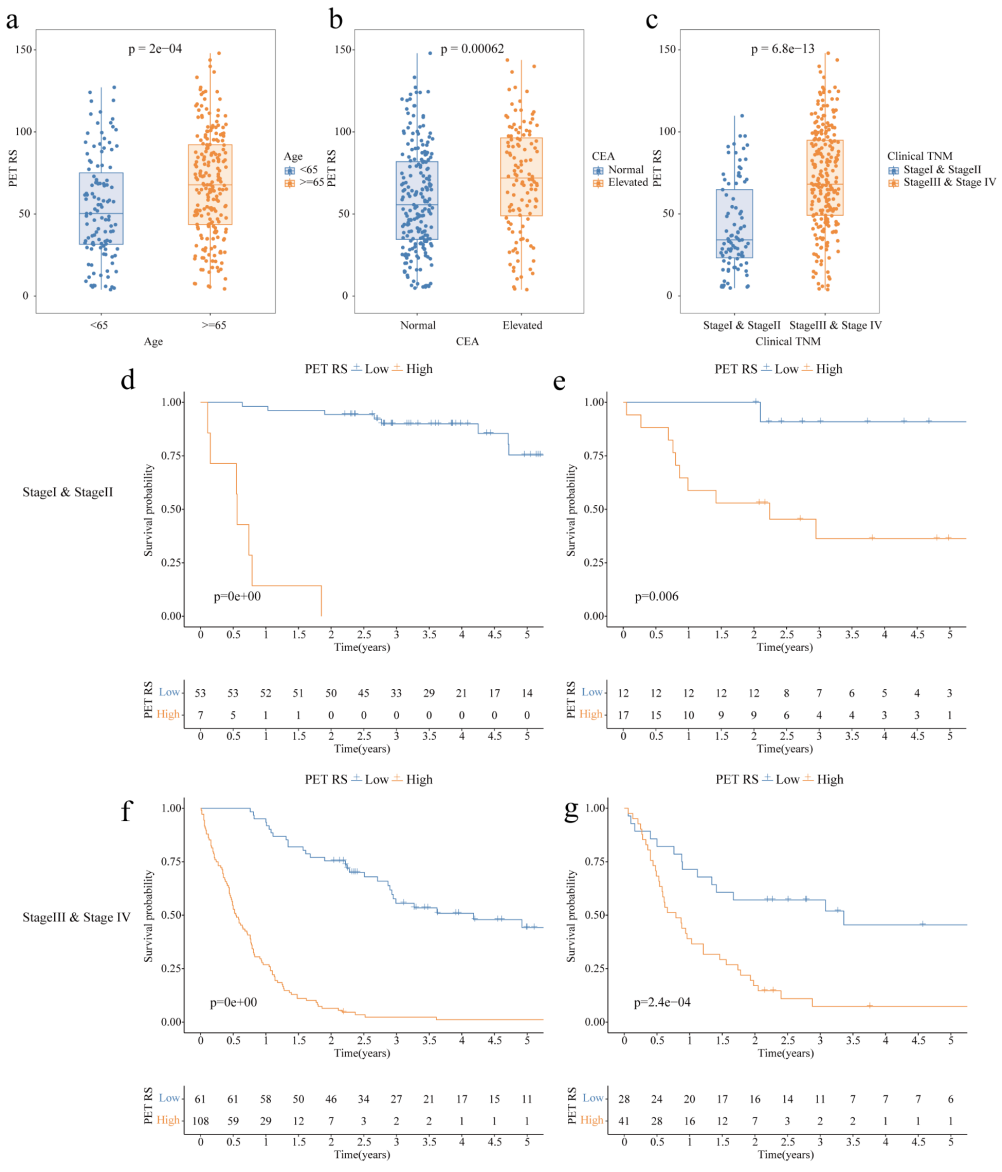
Analysis of correlations between PET RS and clinical features in GC patients; Kaplan-Meier survival analysis of OS in patients with GC with different Clinical TNM stages according to PET RS. (**a**) Comparison of PET RS in GC patients of different ages. (**b**) Comparison of PET RS in GC patients with different CEA. (**c**) Comparison of PET RS in GC patients with different Clinical TNM stages. (**d**) Classification of Stage I and Stage II patients with GC in the training cohort (**e**) Classification of Stage I and Stage II patients with GC in the validation cohort. (**f**) Classification of Stage III and Stage IV patients with GC in the training cohort. (**g**) Classification of Stage III and Stage IV patients with GC in the validation cohort. PET: Positron emission tomography; RS: Radiomics score; GC: Gastric cancer; OS: Overall survival. TNM: Tumor-node-metastasis; CEA: Carcinoembryonic antigen; TNM: Tumor-node-metastasis

Differential analysis of PET RS with different clinical features in all patients showed that older age (*p* < 0.001, Fig. 6a), elevated CEA levels (*p* < 0.001, Fig. 6b), and more advanced clinical TNM stage (*p* < 0.001, Fig. 6c) were associated with higher PET RS. We performed a stratified analysis of clinical TNM in the two cohorts, and the results showed that PET RS could better differentiate the survival of patients at different stages of GC (Fig. 6d-g).

## Discussion

Radiomics is a combination of noninvasive imaging and artificial intelligence technologies for applications in the diagnosis, prognosis, and individualized treatment of diseases. Jiang et al. used a CT-based radiomics model to predict disease-free survival (DFS) and OS in GC patients, and their results showed that the radiomic signal was a predictor of DFS and OS [24]. Wang et al. constructed a CT-based radiomic nomogram to predict lymph nodes in GC [25]. Xu et al. applied a machine-learning model with CT to predict the pathological downgrading of neoadjuvant chemotherapy in patients with advanced GC, which contributed to subsequent surgical treatments [26]. However, compared to CT, PET has unique advantages in the differential diagnosis, precise staging, and distant metastasis diagnosis of GC [27], which are conducive to increasing the survival rate of patients. Therefore, we extracted radiological features from PET images, calculated PET RS using LASSO and RSF models, and combined them with other features to construct a nomogram to predict the prognosis of GC in this study. The nomogram showed a better predictive ability in the two cohort.

Previous studies have demonstrated the potential of PET radiomics in the prediction of lymph node and peritoneal metastases [28, 29]. It has also been shown that PET radiomics was a predictor of OS and DFS, as well as the benefit of chemotherapy in patients [30]. Our study also demonstrated that PET RS predicted OS in patients with GC and that GC patients with high PET RS had poorer prognoses for survival. Findlay et al. showed that routine staging of GC using PET/CT could detect metastases and predict early postoperative recurrence [6]. Clinical TNM staging was added to take advantage of PET imaging and provide patients with more accurate clinical TNM staging. The results of this study indicate that clinical TNM staging is a reliable predictor of patient prognosis, which also provides a basis for treatment strategies for patients with advanced GC who cannot undergo invasive surgery. We constructed a nomogram that incorporated clinical TNM, clinical features, and PET RS and compared it with a single clinical feature model and a clinical feature combined with a clinical TNM model. These results suggest that PET RS can provide additional information to a certain extent, which can be obtained from the quantitative assessment of heterogeneity using the radiological features of PET [31]. This may be related to the effect of tumor heterogeneity on survival and prognosis [32, 33]. In other words, PET RS can compensate for the predictive value of clinical TNM, contributing to personalized treatment and patient follow-up. The underlying mechanisms may be accessible at the genomic or histological levels [34].

There are several limitations to this study. This was a single-center study with a small sample size and no external validation. We intend to increase the sample size and to conduct multicenter studies to ensure that the model can be applied to a larger population in subsequent studies. Additionally, most patients selected for PET had advanced GC, which may have led to a potential bias. Furthermore, we only studied PET images, but we plan to construct a combined CT and PET model to make full use of the imaging data in the future.

## Conclusion

In conclusion, we constructed a PET-based radiomics model and combined it with clinical TNM staging and clinical features to predict OS in patients with GC. PET RS can be effective in predicting patients’ clinical outcomes, adding predictive value to clinical TNM staging.

## Data Availability

No datasets were generated or analysed during the current study.

## Abbreviations

GC: Gastric cancer
PET: positron emission tomography
TNM: Tumor-node-metastasis
OS: Overall Survival
18 F-FDG: Fluorine-18-fludeoxyglucose
LASSO: The least absolute shrinkage and selection operator
RSF: Random survival forest
RS: Radiomics scores
CEA: Carcinoembryonic antigen
CT: Computed tomography
SUVmax: Maximum standardized intake value
SUVmean: Mean standardized intake value
MTV: Metabolic tumor volume
TLG: total pathological glycolysis
NRS: 2002 Nutritional risk screening 2002
BMI: Body mass index
CA199: Carbohydrate antigen199
VOI: volume of interest
C-index: The Harrell consistency index
ICC: Intraclass correlation coefficients
DCA: Decision curve analysis
IBS: integrated Brier score
